# Supplementary material for: An interactive ImageJ plugin for semi-automated image denoising in electron microscopy
Source: Nat Commun. 2020 Feb 7;11:771. doi: 10.1038/s41467-020-14529-0 (PMC7005902; doi:10.1038/s41467-020-14529-0)
Supplement: Supplementary file 1 — Supplementary Information [file 41467_2020_14529_MOESM1_ESM.pdf]

Supplementary material for the manuscript entitled: An Interactive ImageJ Plugin for Semi-automated Image Denoising in Electron Microscopy

This document provides supplementary material for the manuscript entitled: An Interactive ImageJ Plugin for Semi-automated Image Denoising in Electron Microscopy. In particular, we provide references to the raw data that was used for the experiments, a reference to our DenoisEM plugin, visual results of different parameter settings and computational performance metrics of all restoration methods in the plugin.

Supplementary Note 1: data availability

The data that was used to generate the results in the manuscript is available to the community on the locations specified below:

- Arabidopsis root tip (figure 3):
  - Raw: http://bioimagingcore.be/DenoisEM/data/arabidopsis-raw.tif
  - Restored: http://bioimagingcore.be/DenoisEM/data/arabidopsis-restored.tif
- Murine heart tissue (figure 4):
  - Raw: http://bioimagingcore.be/DenoisEM/data/murine-raw.tif
  - Restored: http://bioimagingcore.be/DenoisEM/data/murine-denoised.tif
- Arabidopsis root tip – dwell time (figure 5):
  - 4 μs dwell time, 8 nm pixel size: http://bioimagingcore.be/DenoisEM/data/dwelltime/2K_4us_8nm.tif
  - 2 μs dwell time, 8 nm pixel size: http://bioimagingcore.be/DenoisEM/data/dwelltime/2K_2us_8nm.tif
  - 1 μs dwell time, 8 nm pixel size: http://bioimagingcore.be/DenoisEM/data/dwelltime/2K_1us_8nm.tif
  - 1 μs dwell time, 8 nm pixel size + Tikhonov denoising: http://bioimagingcore.be/DenoisEM/data/dwelltime/2K_1us_8nm[Tikhonov].tif
  - 4 μs dwell time, 16 nm pixel size: http://bioimagingcore.be/DenoisEM/data/dwelltime/1K_4us_16nm.tif
- Mouse heart tissue (figure 6):
  - Raw: http://bioimagingcore.be/DenoisEM/data/mouse-raw.tif
  - Restored: http://bioimagingcore.be/DenoisEM/data/mouse-denoised.tif
- CREMI challenge data – fly brain (figure 7):
  - Full dataset: https://www.cremi.org
  - Subset: http://bioimagingcore.be/DenoisEM/data/flybrain/

Supplementary Note 2: Data reproducibility

This section describes detailed information on how to reproduce the results from the manuscript.

Data generation

All the biological samples have been prepared according to the methods section (subsection on sample preparation). Image acquisition was performed using electron microscopes with acquisition settings as described in the methods section (subsection on image acquisition). The acquired datasets that were used in the experiments are available on `http://bioimagingcore.be/DenoisEM/data/`.

Image denoising

The pseudocode for the noise/blur estimation, parameter estimation and denoising algorithms is described in the methods section (subsection on implemented restoration methods). The actual implementations of the noise/blur estimation and denoising algorithms are provided in the DenoisEM code repository (`https://github.com/vibbits/EMDenoising/tree/master/src/main/quasar`). The implementation of our linear regression for parameter estimation is provided as supplementary code. The used parameter settings for each experiment are provided in the methods section (subsection on image restoration settings for the experiments).

Experiment specifics

- The first experiment in the main paper (section on improved visualization of 3D EM ultrastructure) requires noise estimation[1]. The implementation of this algorithm is provided in the DenoisEM code repository (`https://github.com/vibbits/EMDenoising/blob/master/src/main/quasar/estimate_noise.q`). However, this noise estimator is currently not stable enough to use as default in the plugin.

- The experiment in subsection on increased throughput of 3D EM imaging employs spectral analysis, we have used the Matlab implementation.

- The experiment in subsection on improved segmentation quality and faster image analysis involves cell counting with the Cell Counter plugin from ImageJ (`https://imagej.nih.gov/ij/plugins/cell-counter.html`). The Analyze Particles tool was used as an alternative and is available on `https://imagej.nih.gov/ij/docs/menus/analyze.html`. The second part of this experiment involves the CREMI dataset (available on `https://cremi.org/`). Segmentation of the membranes was performed using ilastik (`https://www.ilastik.org/`).

- The experiment in subsection “Performance at low latency and easily extensible” discusses the computational performance of the denoising algorithms implemented in Quasar, compared to Matlab and (Java based) ImageJ implementations. The Quasar implementations are available in the DenoisEM repository, whereas te Matlab and ImageJ implementations are referred to in the corresponding citations[2–14].

Supplementary Note 3: software

The DenoisEM plugin can be downloaded at our project page http://bioimagingcore.be/DenoisEM. This location additionally provides software and hardware prerequisites, installation instructions, a getting started example and frequently asked questions. We have also provided a user manual, available at http://bioimagingcore.be/DenoisEM/doc/DenoisEM-manual.pdf, where the user can find practical information about the plugin for *e.g.* parameter finetuning. DenoisEM is open source software available through https://github.com/vibbits/EMDenoising. We are happy to help with any practical issues regarding the plugin and stimulate the community to contact us for this (denoisem@irc.vib-ugent.be).

Supplementary Note 4: Java-Quasar bridge

Overview

The Java-Quasar bridge is a software module that constitutes the interface between the DenoisEM plugin for ImageJ and the Quasar runtime engine. The DenoisEM plugin provides the user interface for the various denoising algorithms, whereas Quasar is the software abstraction layer that handles all aspects of large-scale parallel computation of the denoising algorithms on the GPU (via CUDA or OpenCL), or the CPU (via OpenMP).

Since DenoisEM is an ImageJ plugin that must be implemented in Java, but Quasar offers a C++ application programming interface (API), the Java-Quasar bridge is implemented in Java as well, but communicates with Quasar via the Java Native Interface (JNI). The Java-Quasar bridge provides a concise set of simple yet intuitive Java wrapper objects around equivalent Quasar objects that are allocated and operated on by the Quasar runtime library. These wrapper objects can represent both data (such as images) and code (such as functions implemented in the high-level Quasar programming language). The wrapper objects provide abstractions that shield the Java programmer from the complexity of parallel programming on the GPU. They typically consist mainly of so-called Java native functions that are implemented in the C++ programming language, and which are bundled in a dynamically linked library (DLL) which is loaded at runtime by the Java-Quasar bridge. This architecture is schematically shown in Supplementary Figure 17.

Wrapper objects

The most important wrapper objects provided by the Java-Quasar bridge, and their responsibility:

- The `JavaQuasarBridge` class handles initialization of the Java-Quasar bridge. Its essential role is to load the JNI native library (.dll) that implements the Java object wrappers, and to start up the Quasar runtime engine.
- The `QHost` class represents the Quasar runtime. It has functionality to start and stop the Quasar runtime engine, and to load Quasar modules (a set of functions). Such Quasar modules can be either pre-compiled Quasar code, or they can the source code for functions implemented in the Quasar programming language. In the latter case they will be compiled at runtime into a binary format which can transferred to, and executed on the graphics card.
- A `QValue` object represents a dynamically typed variable in Quasar, such as a scalar value or a multi-dimensional array such as an image.
- A `QFunction` object can wrap any Quasar function. Calling this function object from Java with a number of `QValues` as arguments will effectively push the data represented by the `QValue` objects through the JNI interface into the Quasar runtime engine. The Quasar runtime will then autonomously schedule memory transfers between host memory and GPU memory, will ensure that the compiled function code is present on the GPU, will instruct the GPU to execute the function on the `QValue` data, and finally, will transfer the result back to host memory where it is accessible from the Java world.

Thread safety

One common characteristic of low-level libraries such as CUDA is that they can only be called from within a single thread. Since the Java-Quasar bridge – via Quasar – indirectly communicates with CUDA, it too, must respect this requirement. To facilitate this, the Java-Quasar bridge offers the `QExecutor` helper object. It is a Java Executor singleton object which provides a single thread. All code that interacts with Quasar must be executed on this one thread.

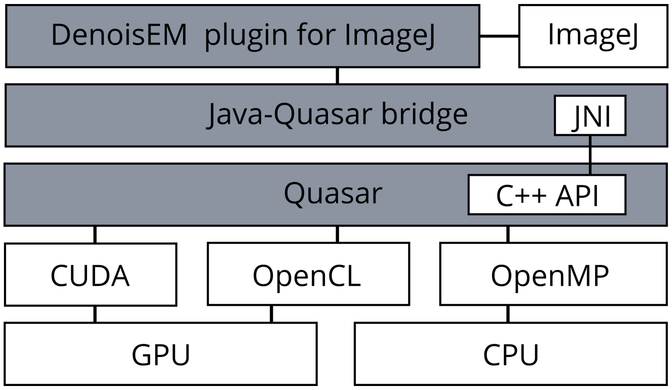

Supplementary Figure 1. DenoisEM software architecture

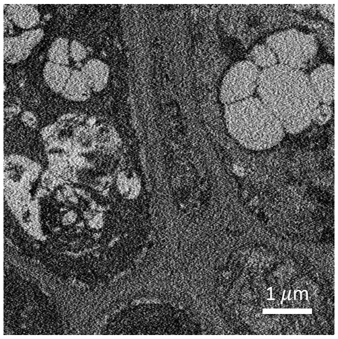

(a) Original noisy image.

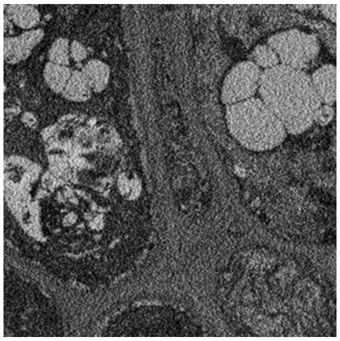

(b) Gaussian filter, $\sigma = 1.0$

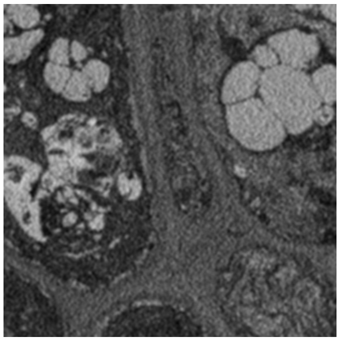

(c) Gaussian filter, $\sigma = 2.0$

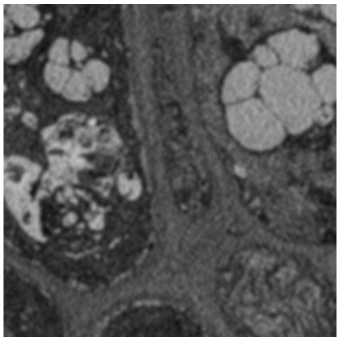

(d) Gaussian filter, $\sigma = 3.0$

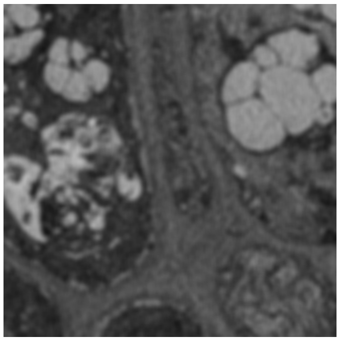

(e) Gaussian filter, $\sigma = 4.0$

Supplementary Figure 2. Denoising with Gaussian filtering. The original noisy image is shown on the left; on the right are the denoised results for increasing values of the blur kernel size σ. As σ grows larger, the Gaussian filter reduces noise more aggressively at the risk of blurring edges.

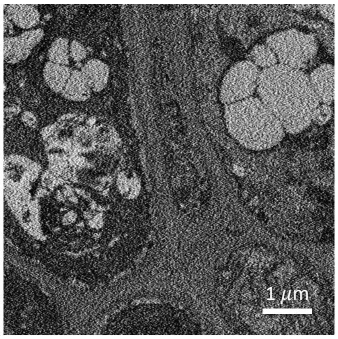

(a) Original noisy image

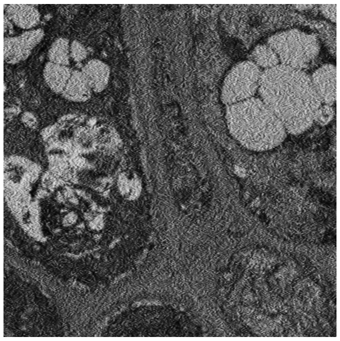

(b) Wavelet thresholding, $T = 0.25$

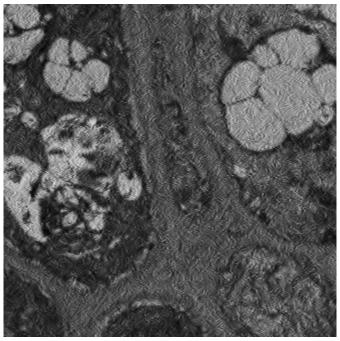

(c) Wavelet thresholding, $T = 0.5$

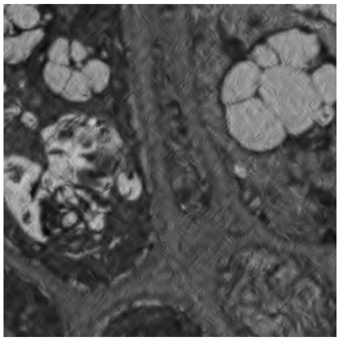

(d) Wavelet thresholding, $T = 1$

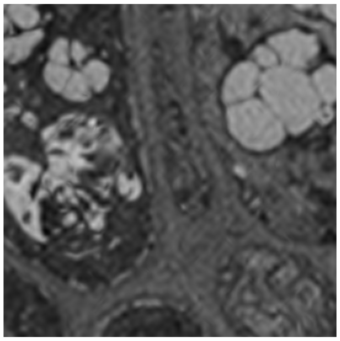

(e) Wavelet thresholding, $T = 2$

Supplementary Figure 3. Denoising via wavelet thresholding. The original noisy image is shown on the left; on the right are the denoised results for increasing values of the wavelet threshold parameter T. Increasing the thresholding parameter leads to higher noise suppression but also potential introduction of wavelet artifacts (*e.g.* ringing).

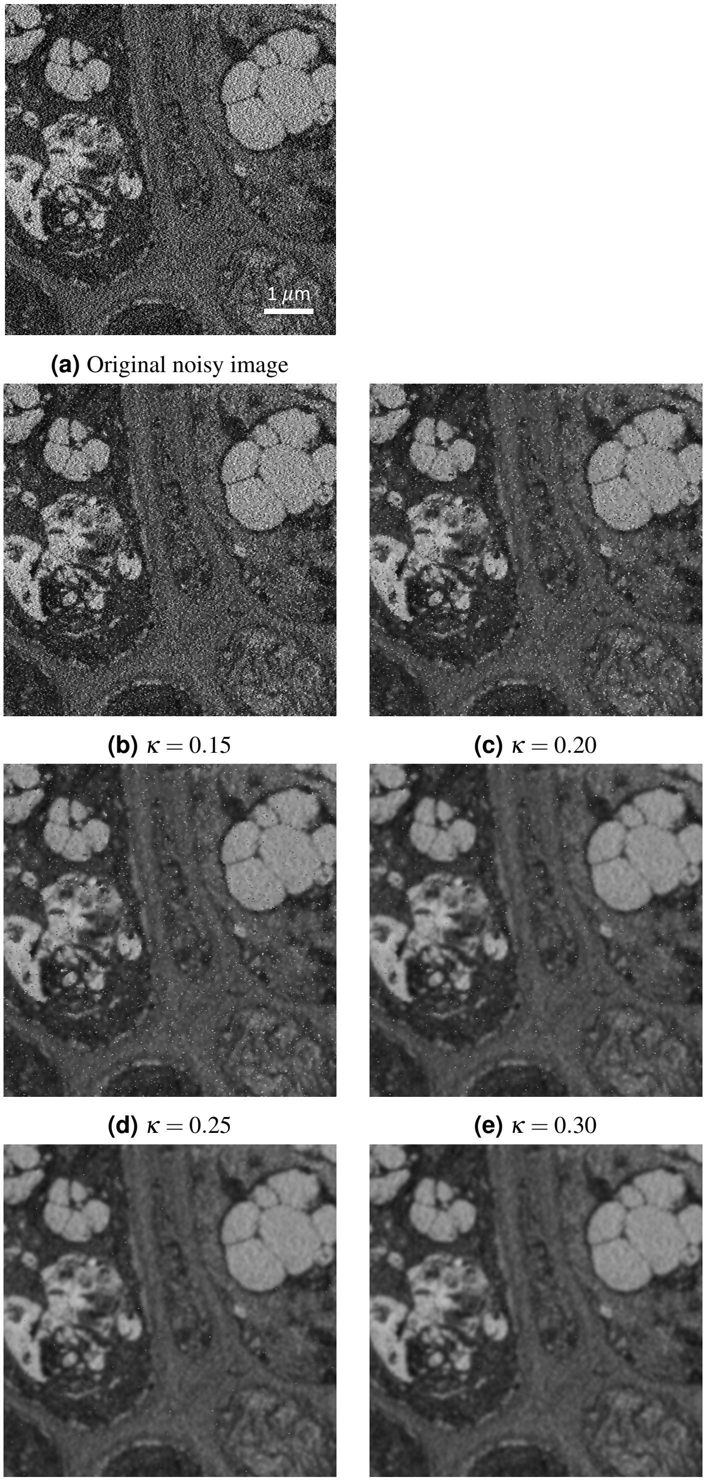

(a) Original noisy image

(b) $\kappa = 0.15$

(c) $\kappa = 0.20$

(d) $\kappa = 0.25$

(e) $\kappa = 0.30$

(f) $\kappa = 0.35$

(g) $\kappa = 0.40$

Supplementary Figure 4. Anisotropic Diffusion denoising. The top image is the original noisy image; the rows below show the result of denoising with anisotropic diffusion for increasing values of the diffusion parameter κ. In all denoised images, the step size was 0.05 and the number of iterations 40. A higher diffusion parameter value leads to higher noise suppression and potentially edge blurring.

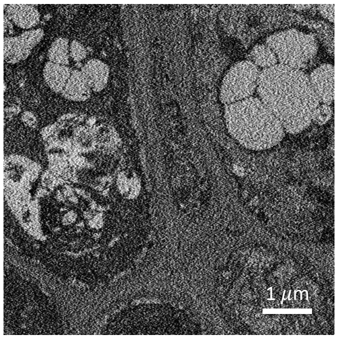

(a) Original noisy image

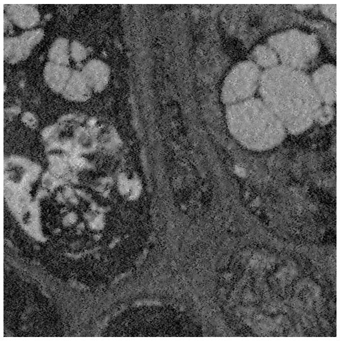

(b) $\sigma_{int} = 1$, $\sigma_{sp} = 3$

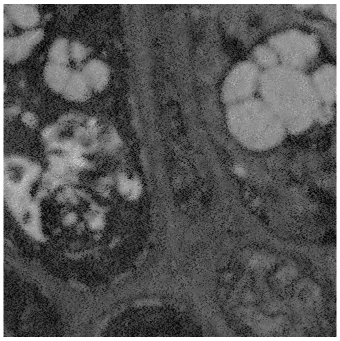

(c) $\sigma_{int} = 1$, $\sigma_{sp} = 5$

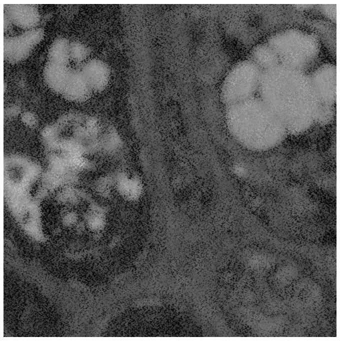

(d) $\sigma_{int} = 1$, $\sigma_{sp} = 7$

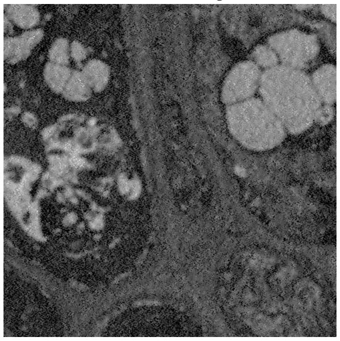

(e) $\sigma_{int} = 4$, $\sigma_{sp} = 3$

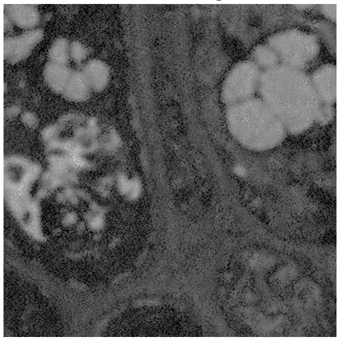

(f) $\sigma_{int} = 4$, $\sigma_{sp} = 5$

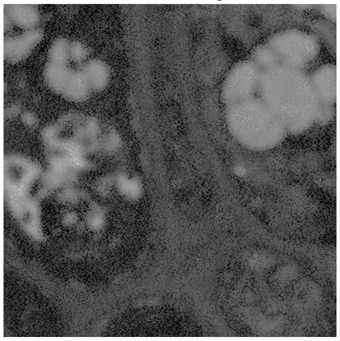

(g) $\sigma_{int} = 4$, $\sigma_{sp} = 7$

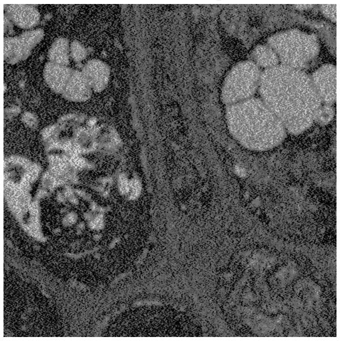

(h) $\sigma_{int} = 7$, $\sigma_{sp} = 3$

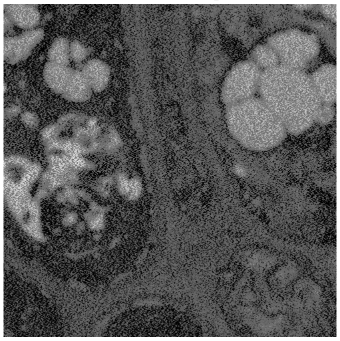

(i) $\sigma_{int} = 7$, $\sigma_{sp} = 5$

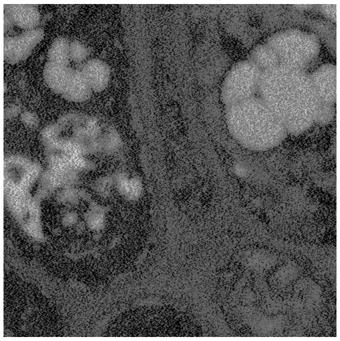

(j) $\sigma_{int} = 7$, $\sigma_{sp} = 7$

Supplementary Figure 5. Bilateral filtering. The top image is the original noisy image; the images underneath show the denoising behavior of the bilateral filter for 9 different combinations of spatial and intensity (range) damping factors: $\sigma_{sp} = 3, 5, 7$ and $\sigma_{int} = 1, 4, 7$. The parameter σ_{sp} has the most significant influence on noise suppression, whereas σ_{int} regularizes the intensity similarity between pixels that can be averaged.

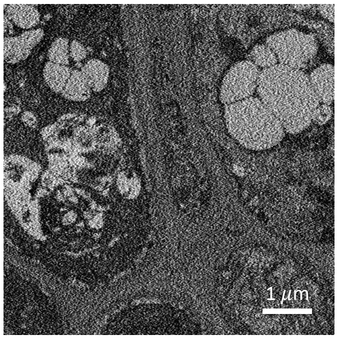

(a) Original noisy image

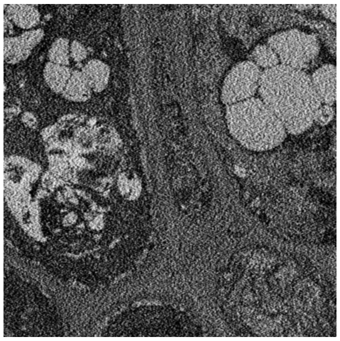

(b) $\lambda = 0.1$, no deconvolution

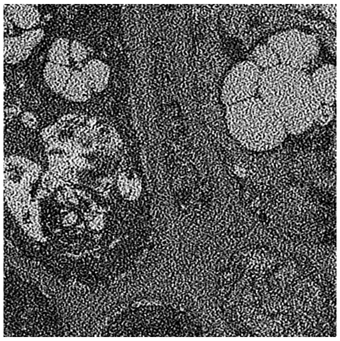

(c) $\lambda = 0.1$, deconvolution

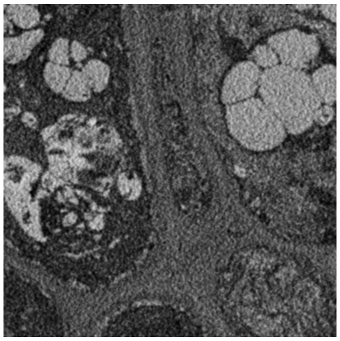

(d) $\lambda = 1$, no deconvolution

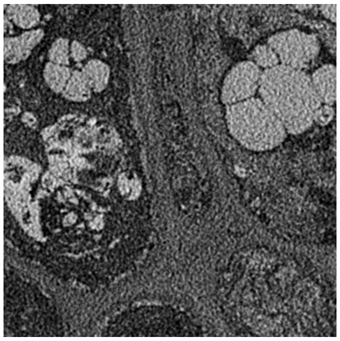

(e) $\lambda = 1$, deconvolution

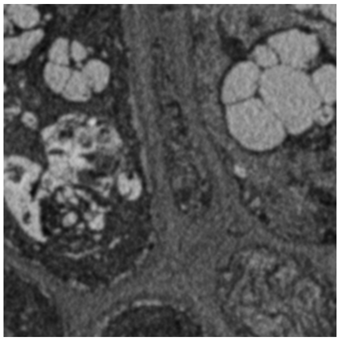

(f) $\lambda = 10$, no deconvolution

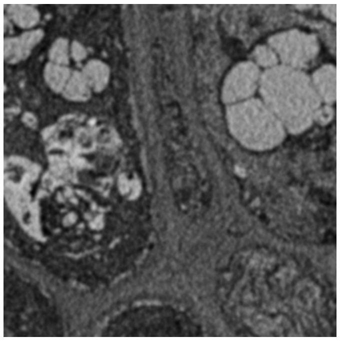

(g) $\lambda = 10$, deconvolution

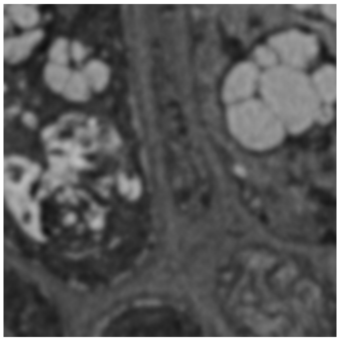

(h) $\lambda = 100$, no deconvolution

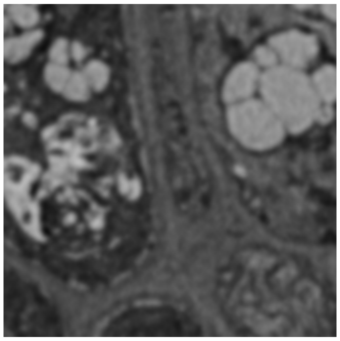

(i) $\lambda = 100$, deconvolution

Supplementary Figure 6. Tikhonov denoising, without deconvolution (middle column) and with deconvolution (right column). In all images the number of iterations was 100. For the images where deconvolution was applied, the blur kernel standard deviation had $\sigma = 1.5$. The λ parameter offers control over the degree of noise suppression and deconvolution slightly sharpens the result.

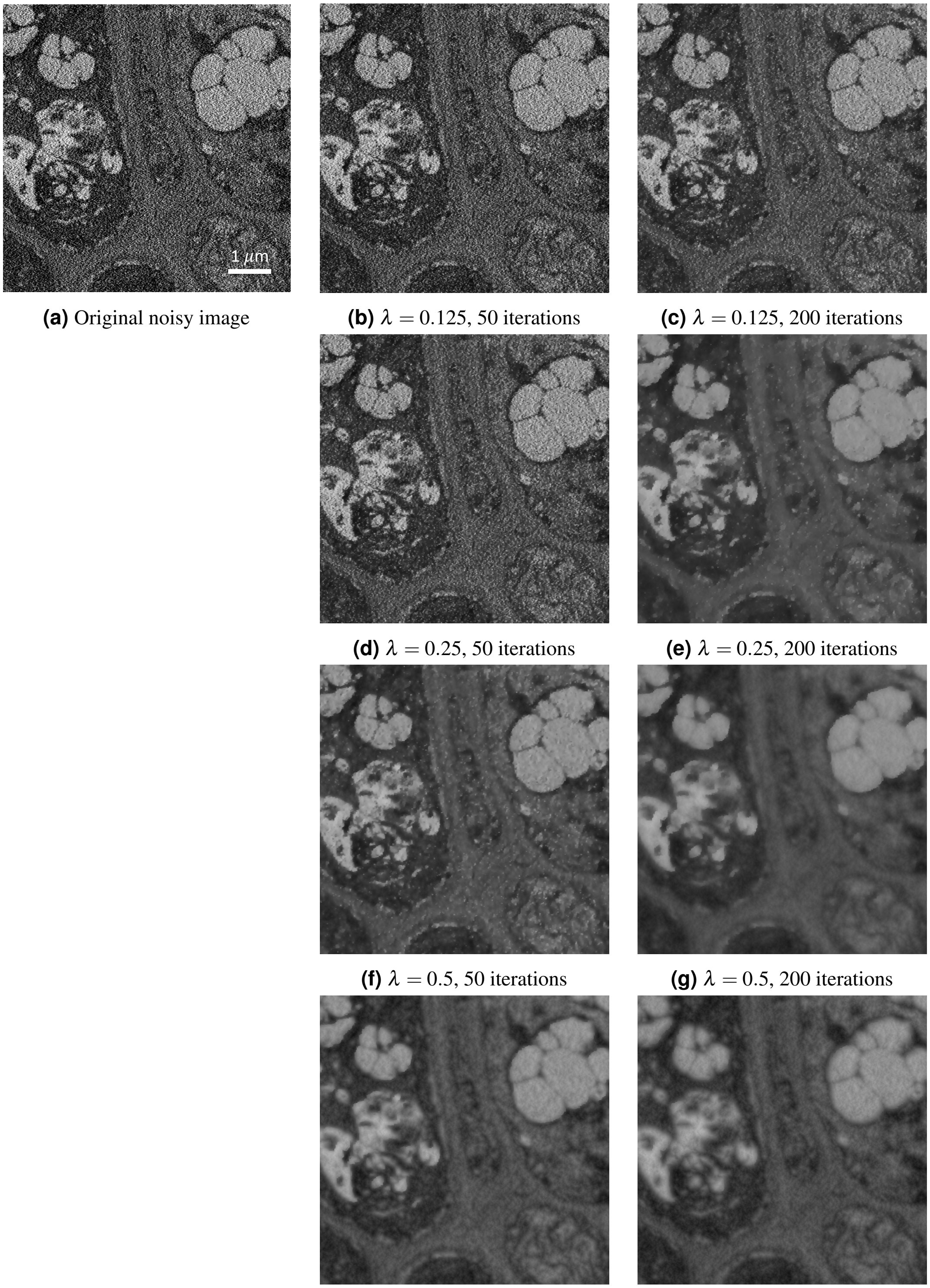

(a) Original noisy image

(b) $\lambda = 0.125$, 50 iterations

(c) $\lambda = 0.125$, 200 iterations

(d) $\lambda = 0.25$, 50 iterations

(e) $\lambda = 0.25$, 200 iterations

(f) $\lambda = 0.5$, 50 iterations

(g) $\lambda = 0.5$, 200 iterations

(h) $\lambda = 1$, 50 iterations

(i) $\lambda = 1$, 200 iterations

Supplementary Figure 7. Total Variation denoising. On the left is the original noisy image. From top to bottom are the denoised results for increasing values of the regularization parameter λ. The middle column used 50 iterations of the algorithm, the right column shows a more precise result using 200 iterations. The λ parameter offers control over the degree of noise suppression and the amount of iterations is preferably as high as possible in order to guarantee convergence.

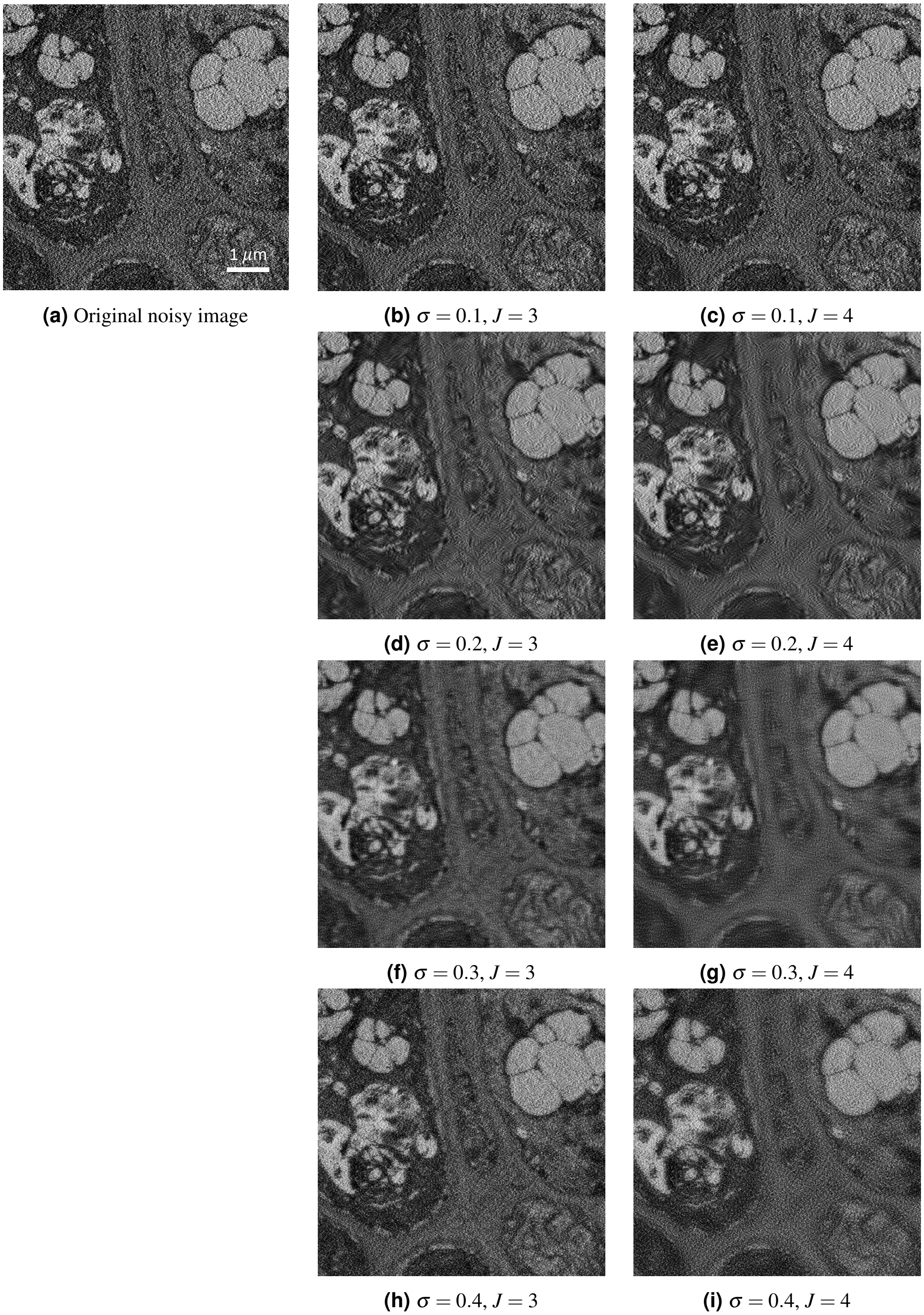

(a) Original noisy image
(b) $\sigma = 0.1, J = 3$
(c) $\sigma = 0.1, J = 4$
(d) $\sigma = 0.2, J = 3$
(e) $\sigma = 0.2, J = 4$
(f) $\sigma = 0.3, J = 3$
(g) $\sigma = 0.3, J = 4$
(h) $\sigma = 0.4, J = 3$
(i) $\sigma = 0.4, J = 4$

Supplementary Figure 8. BLS-GSM. The parameter σ preferably matches the noise standard deviation, whereas an increasing number of scales typically leads to higher noise suppression.

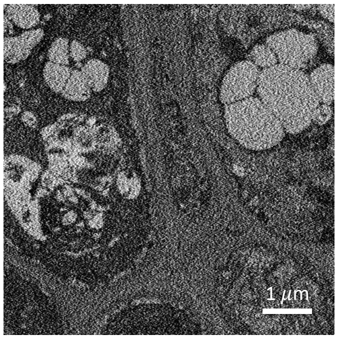

(a) Original noisy image

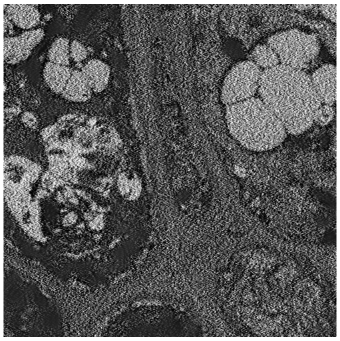

(b) $h = 0.35$, $W = 10$

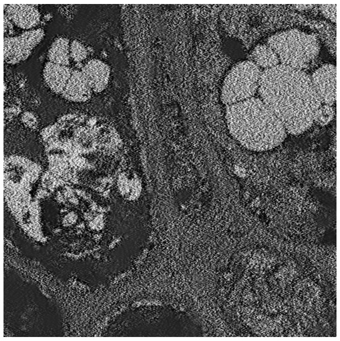

(c) $h = 0.35$, $W = 20$

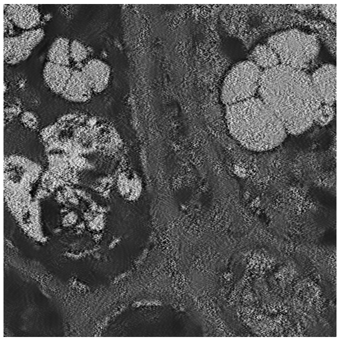

(d) $h = 0.4$, $W = 10$

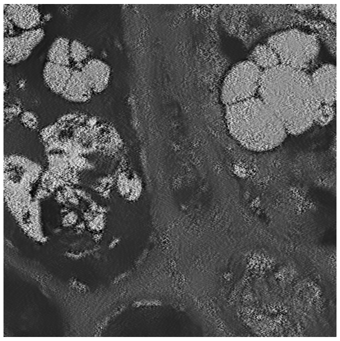

(e) $h = 0.4$, $W = 20$

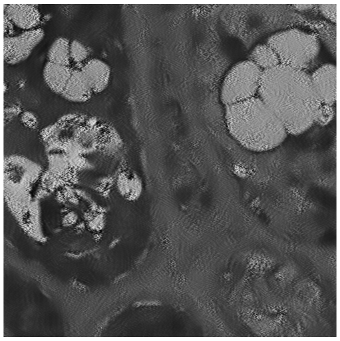

(f) $h = 0.45$, $W = 10$

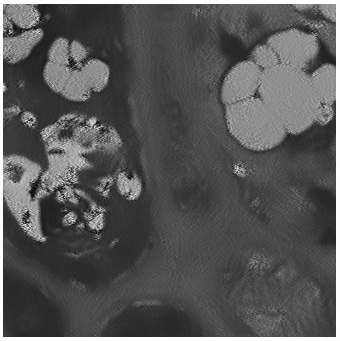

(g) $h = 0.45$, $W = 20$

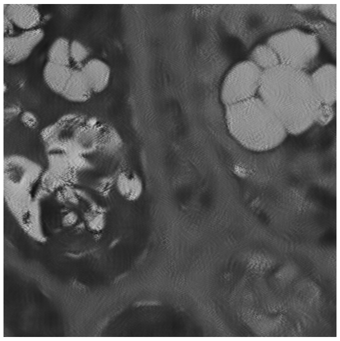

(h) $h = 0.5$, $W = 10$

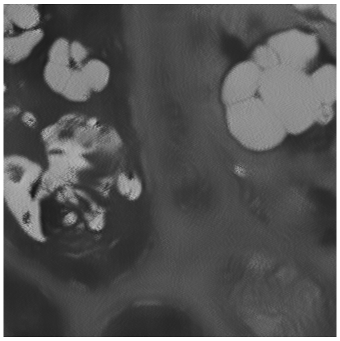

(i) $h = 0.5$, $W = 20$

Supplementary Figure 9. Non-local means denoising without deconvolution. The half block size used for averaging (similarity window) is $B = 7$ (*i.e.* a 15×15 window) in all images. Increasing the damping parameter h leads to more noise suppression. Increasing the search window size leads to potentially better pixel candidates for averaging, but a higher computational cost.

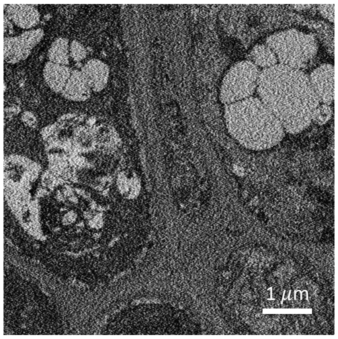

(a) Original noisy image

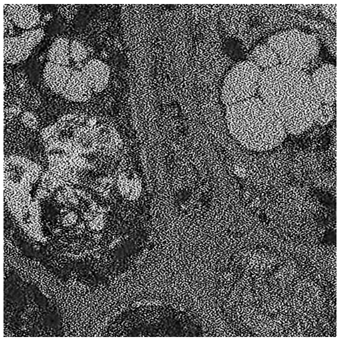

(b) $h = 0.35$, $\lambda = 0.5$

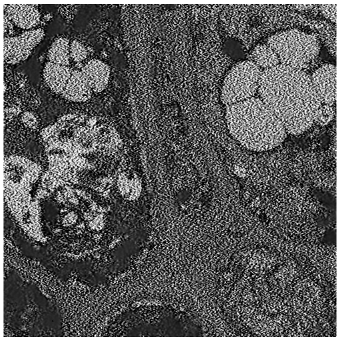

(c) $h = 0.35$, $\lambda = 2$

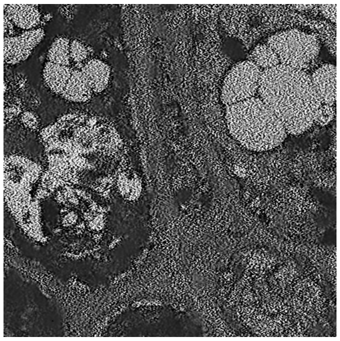

(d) $h = 0.4$, $\lambda = 0.5$

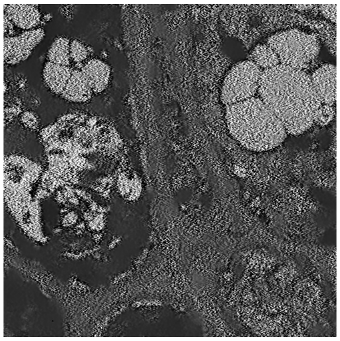

(e) $h = 0.4$, $\lambda = 2$

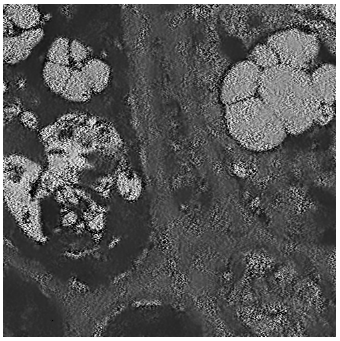

(f) $h = 0.45$, $\lambda = 0.5$

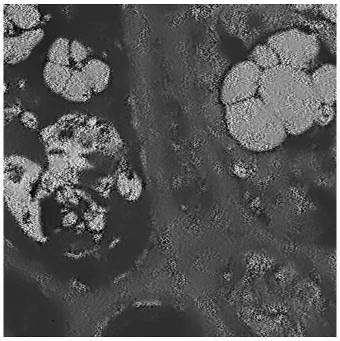

(g) $h = 0.45$, $\lambda = 2$

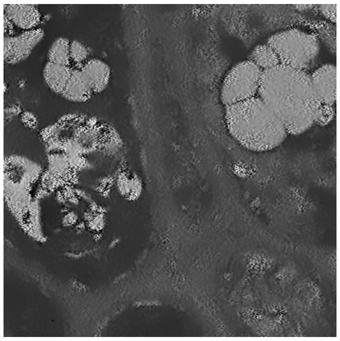

(h) $h = 0.5$, $\lambda = 0.5$

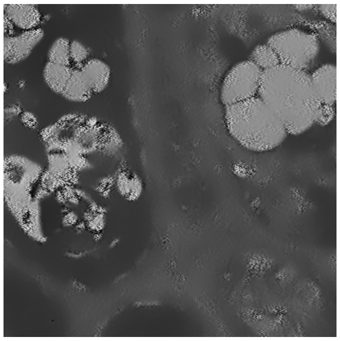

(i) $h = 0.5$, $\lambda = 2$

Supplementary Figure 10. Non-local means denoising with deconvolution. The half block size used for averaging (similarity window) is $B = 7$ (*i.e.* a 15×15 window) in all images. The half search window size is $W = 10$ (*i.e.* a 21×21 window) in all images. Lower values of the regularization parameter λ emphasize deconvolution over denoising. Increasing the damping parameter h leads to more noise suppression as opposed to sharpening.

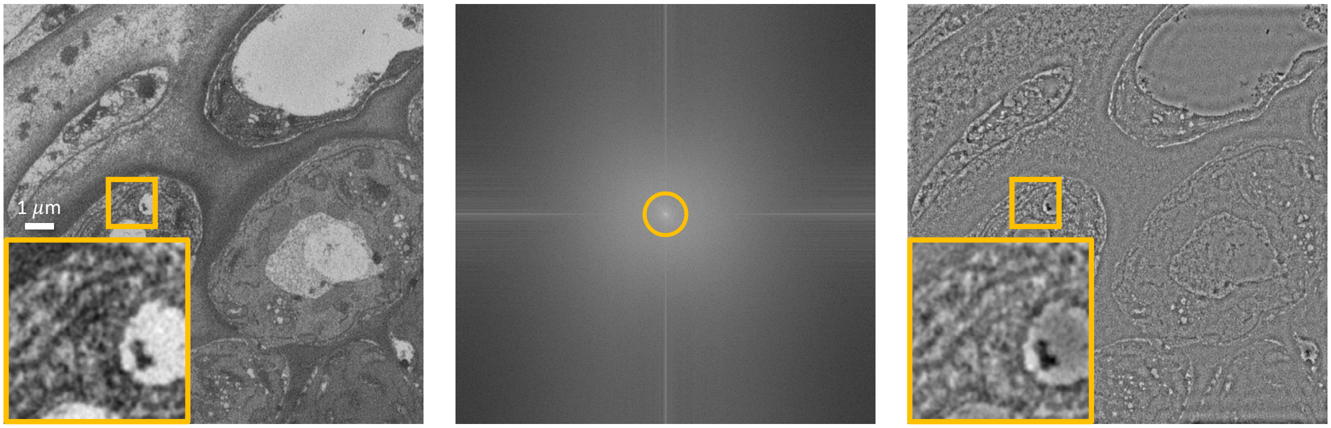

(a) 1 μs dwell time, 8 nm pixels + denoising (b) Frequency spectrum of (a) (c) Masked spectral reconstruction of (b)

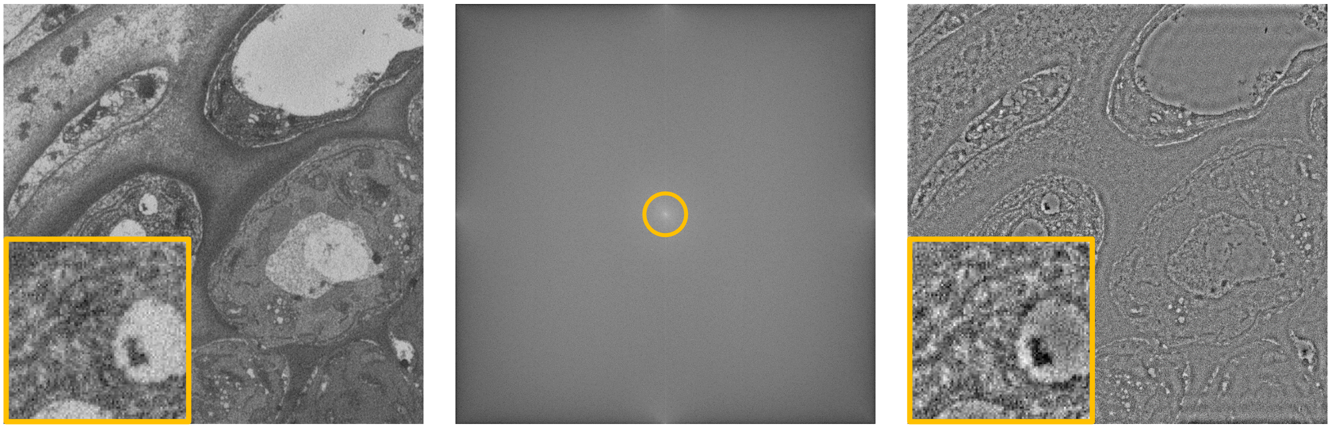

(d) 4 μs dwell time, 16 nm pixels (e) Frequency spectrum of (d) (f) Masked spectral reconstruction of (e)

Supplementary Figure 11. (a) SBF-SEM section acquired at 1 μs dwell-time, 8 nm pixel size and denoised by an expert using DenoisEM, (d) the same image, acquired at 4 μs dwell-time, 16 nm pixel size, which results in approximately the same acquisition time. The corresponding Fourier spectra (b,e) show that our the denoising algorithms do not significantly affect the high-frequency components of the image. When masking out the low-frequency components (indicated by the yellow circle), we can reconstruct the image and see the resolution improvements in the spatial domain (c,f).

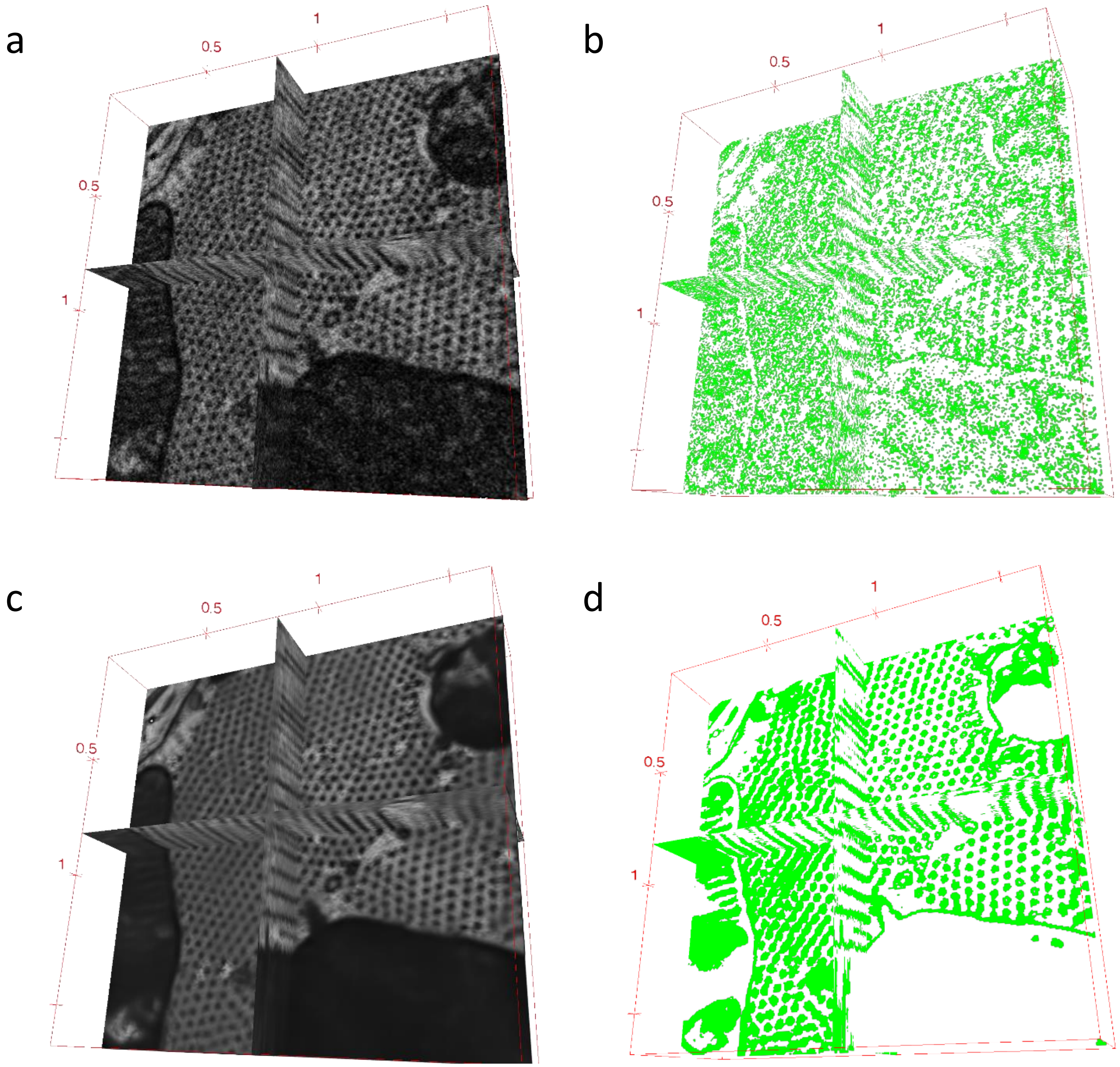

Supplementary Figure 12. (a) A 3D ROI from a FIB-SEM dataset was used for multi-orthogonal visualisation, using ImageJ 3D viewer. (b) A green mask created by applying intensity thresholding is shown in the same manner. (c) Similar visualisation was done on the same ROI, after denoising was applied. (d) Orthogonal views of the denoised data and mask after thresholding of the denoised data is shown.

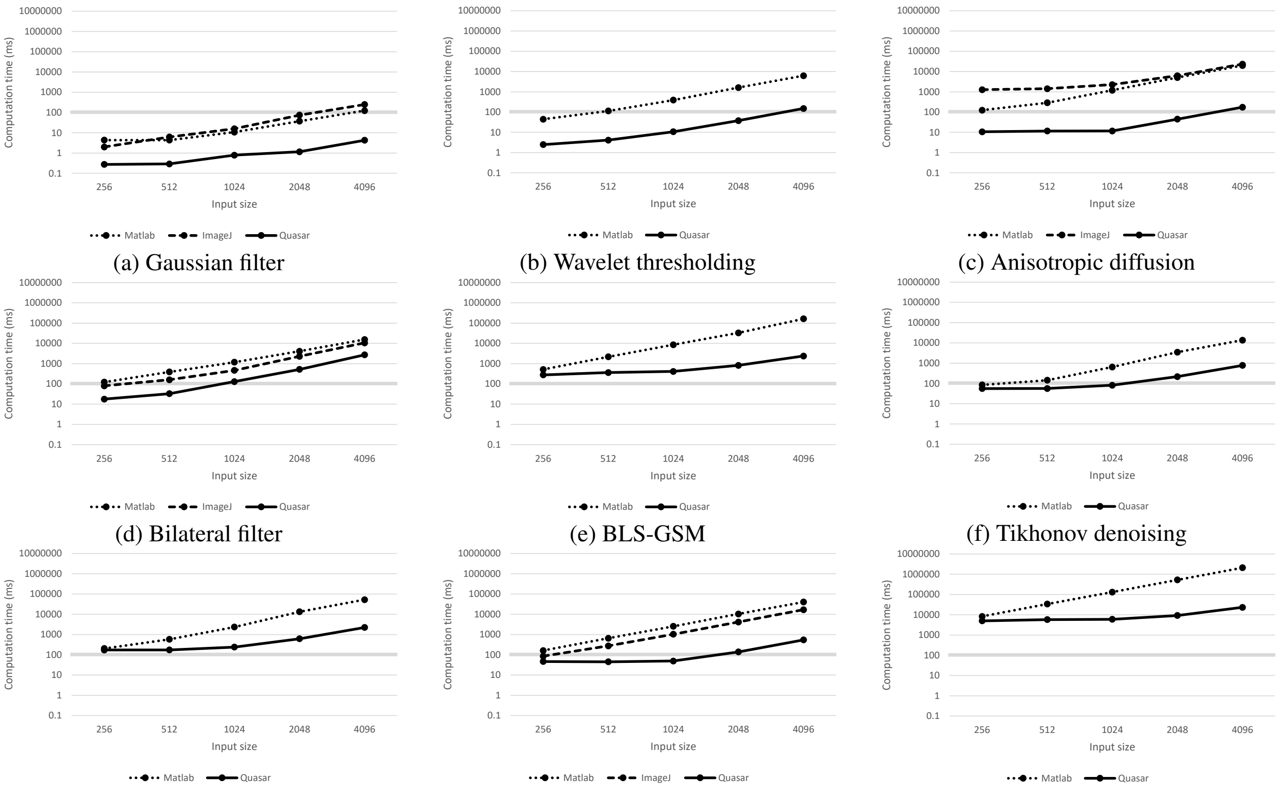

(a) Gaussian filter (b) Wavelet thresholding (c) Anisotropic diffusion

(d) Bilateral filter (e) BLS-GSM (f) Tikhonov denoising

(g) Total variation denoising (h) Non-local means denoising (i) Non-local means deconvolution

Supplementary Figure 13. Computational performance (in milliseconds) of denoising algorithms, as indicated, for different input sizes. A comparison is made between the proposed GPU-based Quasar framework and alternative CPU-based implementations in ImageJ[2–5] and MATLAB[6–14]. For each algorithm, we consider inputs of 256^2, 512^2, 1024^2, 2048^2 and 4096^2 pixels. In general, the Quasar implementation performs one to two orders of magnitude faster compared to the existing software packages. 10 Hz is put as cut-off for real-time performance and indicated with a full line on the graph. Timings were measured using an Intel(R) Core(TM) i7-4930K CPU @ 3.40GHz and NVIDIA GTX 1070 GPU.

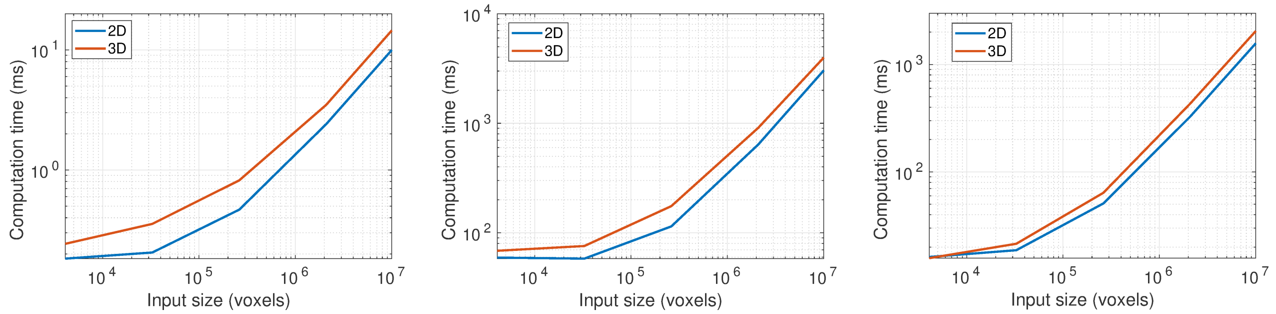

(a) Gaussian filter (b) Bilateral filter (c) Tikhonov denoising

Supplementary Figure 14. Computational performance comparison of 2D and 3D based implementations.

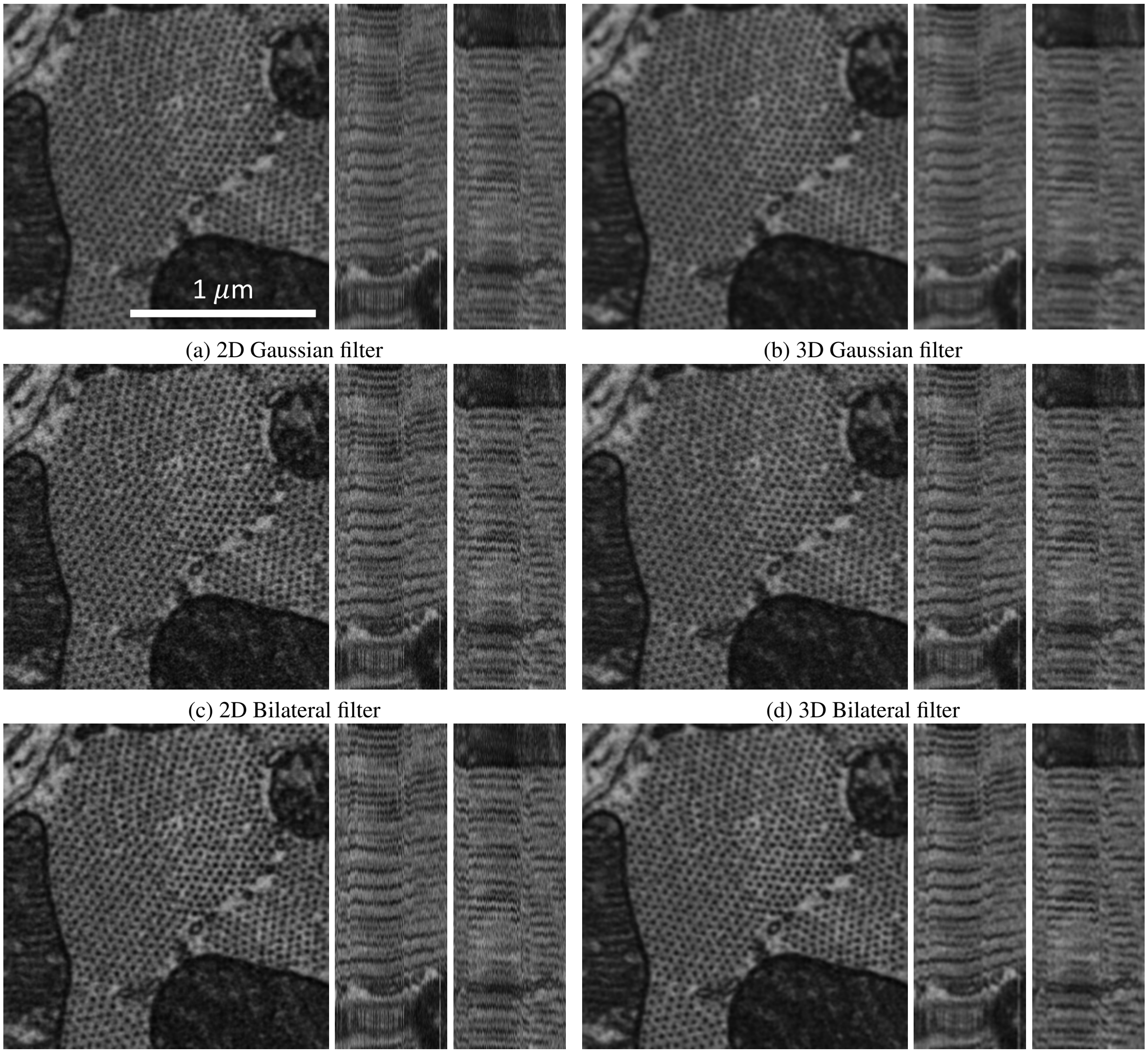

(a) 2D Gaussian filter
(b) 3D Gaussian filter
(c) 2D Bilateral filter
(d) 3D Bilateral filter
(e) 2D Tikhonov denoising
(f) 3D Tikhonov denoising

Supplementary Figure 15. Denoising performance comparison of 2D and 3D based implementations. Each subfigure shows a cross-section along the z, y and x axis (from left to right, respectively).

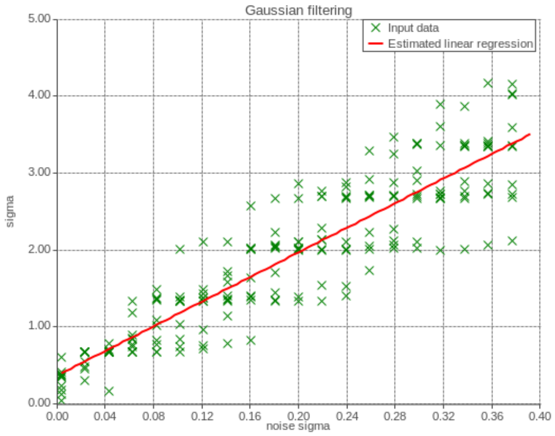

(a) Gaussian filter (σ estimation)

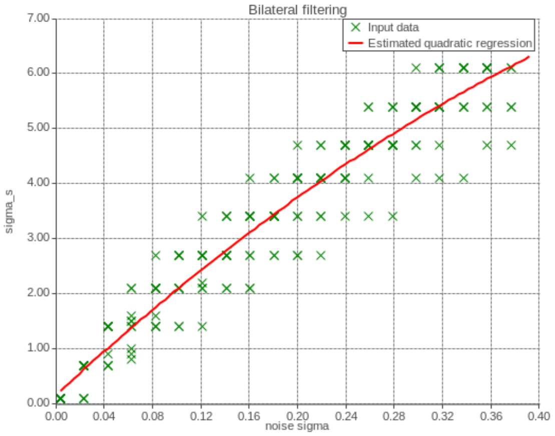

(b) Bilateral filter (σ_s estimation, $\sigma_r = 1$ fixed)

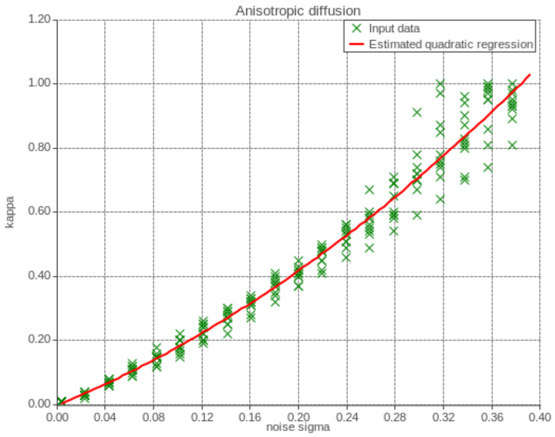

(c) Anisotropic diffusion (κ estimation)

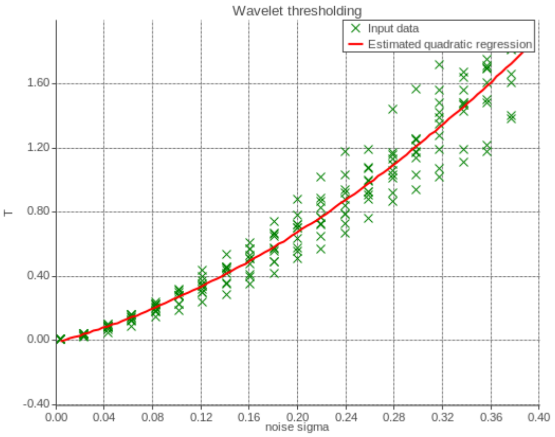

(d) Wavelet thresholding (T estimation)

Supplementary Figure 16. Estimations of the optimal parameter settings based on linear/quadratic regression on the noise level. The remaining methods are in Figure 17.

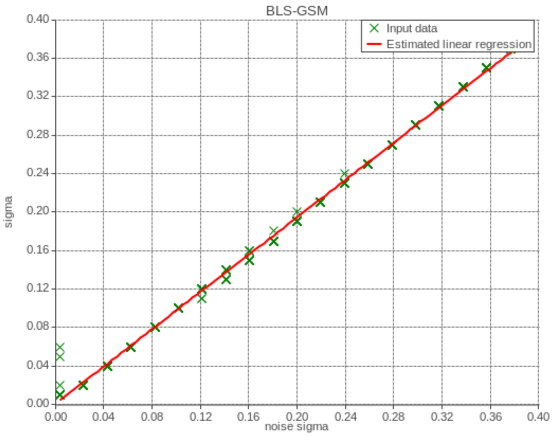

(a) BLS-GSM (σ estimation)

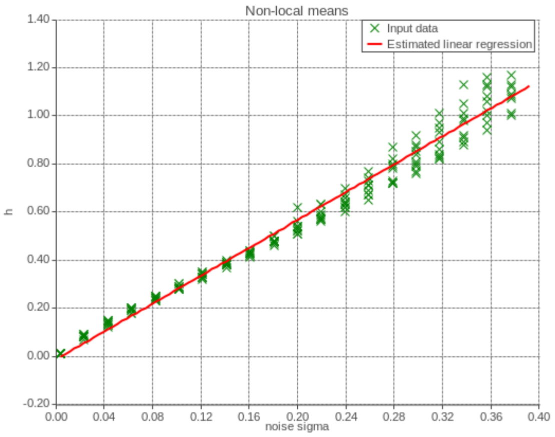

(b) Non-local means (h estimation)

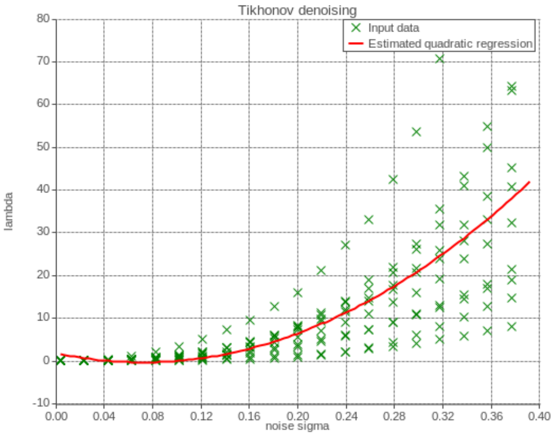

(c) Tikhonov denoising (λ estimation)

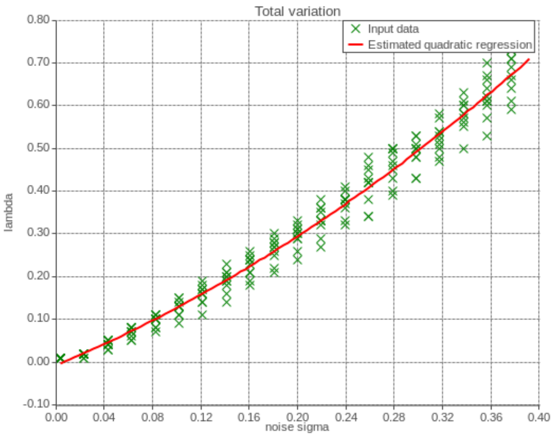

(d) Total variation (λ estimation)

Supplementary Figure 17. Estimations of the optimal parameter settings based on linear/quadratic regression on the noise level. The remaining methods are in Figure 16.

Supplementary References

1. Liu, X., Tanaka, M. & Okutomi, M. Noise level estimation using weak textured patches of a single noisy image. In *IEEE International Conference on Image Processing*, 665–668 (2012). DOI 10.1109/ICIP.2012.6466947.
2. Lieng, E. Gaussian filter (ImageJ). URL https://imagej.nih.gov/ij/plugins/gaussian-filter.html.
3. Pilny, V. & Janacek, J. Anisotropic diffusion (ImageJ). URL https://imagej.nih.gov/ij/plugins/anisotropic-diffusion-2d.html.
4. Sage, D. & Chaudhury, K. N. Bilateral filter (ImageJ). URL http://bigwww.epfl.ch/algorithms/bilateral-filter/.
5. Behnel, P. & Wagner, T. Non-local means (ImageJ). URL https://imagej.net/Non_Local_Means_Denoise.
6. Mathworks. Gaussian filter (Matlab). URL https://www.mathworks.com/help/images/ref/imgaussfilt.html.
7. Lopes, D. Anisotropic diffusion (Matlab). URL https://nl.mathworks.com/matlabcentral/fileexchange/14995-anisotropic-diffusion-perona-malik.
8. Chaudhury, K. Bilateral filter (Matlab). URL https://nl.mathworks.com/matlabcentral/fileexchange/56158-fast-and-accurate-bilateral-filtering.
9. Goossens, B. Non-local means (Matlab). URL https://quasar.ugent.be/bgoossen/download_nlmeans/.
10. Portilla, J. BLS-GSM (Matlab). URL https://www.io.csic.es/PagsPers/JPortilla/software/file/3-bls-gsm-image-denoising-toolbox-in-matlab.
11. Cai, S. & Li, K. Wavelet shrinkage (Matlab). URL http://eeweb.poly.edu/iselesni/WaveletSoftware/dt2D.html.
12. Roels, J. Tikhonov denoising (Matlab). URL https://telin.ugent.be/~jbroels/image_restoration/tikhonov/.
13. Roels, J. Total variation denoising (Matlab). URL https://telin.ugent.be/~jbroels/image_restoration/total_variation/.
14. Roels, J. *et al.* Bayesian deconvolution of scanning electron microscopy images using point-spread function estimation and non-local regularization. In *International Conference of the IEEE Engineering in Medicine and Biology Society*, vol. 2016-Octob, 443–447 (2016). DOI 10.1109/EMBC.2016.7590735.
