# Supplementary material for: An interactive ImageJ plugin for semi-automated image denoising in electron microscopy
Source: Nat Commun. 2020 Feb 7;11:771. doi: 10.1038/s41467-020-14529-0 (PMC7005902; doi:10.1038/s41467-020-14529-0)
Supplement: Supplementary file 3 — Reporting Summary [file 41467_2020_14529_MOESM3_ESM.pdf]

nature research

Corresponding author(s): Joris Roels
Last updated by author(s): Dec 17, 2019

Reporting Summary

Nature Research wishes to improve the reproducibility of the work that we publish. This form provides structure for consistency and transparency in reporting. For further information on Nature Research policies, see Authors & Referees and the Editorial Policy Checklist.

Statistics

For all statistical analyses, confirm that the following items are present in the figure legend, table legend, main text, or Methods section.

n/a	Confirmed	
☒	☐	The exact sample size (*n*) for each experimental group/condition, given as a discrete number and unit of measurement
☒	☐	A statement on whether measurements were taken from distinct samples or whether the same sample was measured repeatedly
☒	☐	The statistical test(s) used AND whether they are one- or two-sided<br>*Only common tests should be described solely by name; describe more complex techniques in the Methods section.*
☒	☐	A description of all covariates tested
☒	☐	A description of any assumptions or corrections, such as tests of normality and adjustment for multiple comparisons
☒	☐	A full description of the statistical parameters including central tendency (e.g. means) or other basic estimates (e.g. regression coefficient) AND variation (e.g. standard deviation) or associated estimates of uncertainty (e.g. confidence intervals)
☒	☐	For null hypothesis testing, the test statistic (e.g. *F*, *t*, *r*) with confidence intervals, effect sizes, degrees of freedom and *P* value noted<br>*Give P values as exact values whenever suitable.*
☒	☐	For Bayesian analysis, information on the choice of priors and Markov chain Monte Carlo settings
☒	☐	For hierarchical and complex designs, identification of the appropriate level for tests and full reporting of outcomes
☒	☐	Estimates of effect sizes (e.g. Cohen's *d*, Pearson's *r*), indicating how they were calculated

Our web collection on statistics for biologists contains articles on many of the points above.

Software and code

Policy information about availability of computer code

Data collection	The data was acquired using Serial Block Face and Focused Ion Beam Scanning Electron Microscopes, particularly the Zeiss Merlin (with Gatan 3View2 detector) and Zeiss Crossbeam 540.
Data analysis	Most of the data analysis was performed using the proposed DenoisEM plugin (v1.1.0). This is a plugin for the (Java based) ImageJ framework (we used v1.52) that employs an in-house programming language, called Quasar (available upon request at www.gepura.io). We provide installation instructions, an example demo and an FAQ section on the DenoisEM project page (www.bioimagingcore.be/DenoisEM). The source code of both the Java-Quasar bridge and DenoisEM are provided to the reviewers and are available on Github: respectively https://github.com/vibbits/JavaQuasarBridge and https://github.com/vibbits/EMDenoising. The remaining experiments and/or graphics design were performed using Matlab (v9.5.0.1033004, R2018b), Python 3.7, GIMP (v2.8.22), Inkscape (v0.92.3) and MS Office 2016.

For manuscripts utilizing custom algorithms or software that are central to the research but not yet described in published literature, software must be made available to editors/reviewers. We strongly encourage code deposition in a community repository (e.g. GitHub). See the Nature Research guidelines for submitting code & software for further information.

Data

Policy information about availability of data

All manuscripts must include a data availability statement. This statement should provide the following information, where applicable:

- Accession codes, unique identifiers, or web links for publicly available datasets
- A list of figures that have associated raw data
- A description of any restrictions on data availability

The DenoisEM plugin is available on our webpage (https://bioimagingcore.be/DenoisEM) and figshare (DOI: 10.6084/m9.figshare.9929201). A user manual is provided on the webpage (http://bioimagingcore.be/DenoisEM/user-manual.pdf, DOI: 10.6084/m9.figshare.9929888). The raw data that was used for this manuscript is located on our webpage (https://bioimagingcore.be/DenoisEM/data, DOI: 10.6084/m9.figshare.9929183). The source data underlying Figs 1-9 and

Supplementary Figs 1-15 are provided as a Source Data file (DOI: 10.6084/m9.figshare.9929216).

Field-specific reporting

Please select the one below that is the best fit for your research. If you are not sure, read the appropriate sections before making your selection.

☒ Life sciences ☐ Behavioural & social sciences ☐ Ecological, evolutionary & environmental sciences

For a reference copy of the document with all sections, see nature.com/documents/nr-reporting-summary-flat.pdf

Life sciences study design

All studies must disclose on these points even when the disclosure is negative.

Sample size	For most experiments, the sample size was no crucial factor as the goal was either visualisation or segmentation of specific structures. We validated that regions of just one megavoxel were sufficient. As an exception, the last experiment (containing computational performance figures) involves sample sizes up to 16 megavoxels, which was the largest input size to process within reasonable times (using the CPU based implementations).
Data exclusions	The excluded data (i.e. the data outside the cropped region) was either similar to the cropped region, thereby not significantly influencing the results, or not of interest w.r.t. the research question. These exclusion criteria were pre-established.
Replication	Most experiments involve deterministic algorithms that will yield the same result when repeated (provided the same parameter settings are employed). The remaining algorithms are stochastic (e.g. using random initialisation) and yield similar results when repeated. This has been verified multiple times throughout the experimental workflow.
Randomization	Randomization was not necessary as no statistical analysis was required in our experiments. Our plugin is designed for any type of sample and any type of 3D electron micrograph. We therefore provide maximal variance in the samples (both plant and mouse are represented) and modalities (we consider serial section TEM, SBF-SEM and FIB-SEM).
Blinding	The data acquisition on the one hand and the data analysis on the other were performed by different research experts. Moreover, these experts used deterministic protocols. The investigators were blinded to group allocation during data collection and/or data analysis.

Reporting for specific materials, systems and methods

We require information from authors about some types of materials, experimental systems and methods used in many studies. Here, indicate whether each material, system or method listed is relevant to your study. If you are not sure if a list item applies to your research, read the appropriate section before selecting a response.

Materials & experimental systems

n/a	Involved in the study	
☒	☐	Antibodies
☒	☐	Eukaryotic cell lines
☒	☐	Palaeontology
☐	☒	Animals and other organisms
☒	☐	Human research participants
☒	☐	Clinical data

Methods

n/a	Involved in the study	
☒	☐	ChIP-seq
☒	☐	Flow cytometry
☒	☐	MRI-based neuroimaging

Animals and other organisms

Policy information about studies involving animals; ARRIVE guidelines recommended for reporting animal research

Laboratory animals	C57BL/6 wild type female mouse of 8 weeks old
Wild animals	The study did not involve wild animals
Field-collected samples	The study did not involve field-collected samples
Ethics oversight	According to local ethical regulations of the Flemish universities, no specific approval is required for sacrificing animals for dissections, without being used for animal experiments.

Note that full information on the approval of the study protocol must also be provided in the manuscript.
